# Supplementary material for: Non-invasive vagus nerve stimulation boosts mood recovery after effort exertion
Source: Psychol Med. 2021 Feb 15;52(14):3029–39. doi: 10.1017/S0033291720005073 (PMC9693679; doi:10.1017/S0033291720005073)
Supplement: Supplementary file 1 [file S0033291720005073sup001.docx]

# Supporting Information

**Non-invasive vagus nerve stimulation boosts mood recovery after effort exertion**

Magdalena Ferstl^1^, Vanessa Teckentrup^1^, Wy Ming Lin^1^, Franziska Kräutlein^1^, Anne Kühnel^1,2^, Johannes Klaus^1^, Martin Walter^1,3-4^, & Nils B. Kroemer^1*^

^1^ Department of Psychiatry and Psychotherapy, University of Tübingen, Tübingen, Germany

^2^ Department of Translational Research in Psychiatry, Max Planck Institute of Psychiatry and International Max Planck Research School for Translational Psychiatry (IMPRS-TP), Munich, Germany

^3^ Otto-von-Guericke University Magdeburg, Department of Psychiatry and Psychotherapy, Germany

^4^ Department of Psychiatry and Psychotherapy, University Hospital Jena, Jena, Germany

**Corresponding author***

Dr. Nils B. Kroemer, nils.kroemer@uni-tuebingen.de

Calwerstr. 14, 72076 Tübingen, Germany

## R code used to estimate the hierarchical Bayesian model

brm(formula = Rating | trunc(lb = 0, ub = 100) ~ Stim * fRun + (1 + Stim * fRun |ID),

data = pos_d_TUE1, family = gaussian(),

cores = 4,

prior = c(set_prior("normal(50,15)", class = "Intercept"),

set_prior("normal(0,10)", class = "b"),

set_prior("cauchy(0,5)", class = "sd"),

set_prior("lkj(2)", class = "cor")),

warmup = 1000, iter = 4000, chains = 4, control = list(adapt_delta = 0.8),

sample_prior = TRUE,

save_all_pars = TRUE)

where Stim encodes the stimulation condition (sham vs. taVNS), fRun encodes the run as a factorial variable (Run 1, Run 2, Run 3), ID encodes the participant, and data includes only the positive ratings collected in the study. We ran the same model on the negative ratings with a lower prior on the intercept, but the models did not converge reliably across chains.
